# Trends in the Receipt of Consistent Hospice Professional Visits at the End of Life and Ratings of Hospice Care Quality

**DOI:** 10.1093/geroni/igaa057.228

**Published:** 2020-12-16

**Authors:** Thomas Christian, Joan Teno, Kyle Cobb, Sara Galantowicz, Alan Levitt, Cindy Massuda

**Affiliations:** 1 Abt Associates, Cambridge, Massachusetts, United States; 2 Oregon Health & Science University, Portland, Oregon, United States; 3 Abt Associates, Cambridge, United States; 4 Centers for Medicare & Medicaid Services, Baltimore, United States

## Abstract

Caregivers have identified consistent visits by professional hospice staff at the end of life as positively affecting experiences of care quality. Little is known about the prevalence of such visits. Using 100% Medicare hospice claims with discharge dates in Federal Fiscal Year 2018, we identified the rates of providing skilled nurse or social worker visits to hospice beneficiaries in at least two of the last three days of life and compared these rates to percentages of caregivers indicating they would “definitely” recommend the hospice and caregivers rating the hospice a 9 or 10 on a 10-scale from CAHPS Hospice scores. Among our analytic cohort of 762,238 hospice discharges, 509,585 individuals (66.9%) were visited by a nurse or social worker in at least two of the last three days of life. Beneficiaries lacking these visits were more likely to be black (black 39.6% vs. white 32.2%; AOR 1.32 95% CI 1.29-1.34) or resided in a nursing facility (nursing facility 37.7% vs. patient’s home 32.1%, AOR 1.39 95% CI 1.36-1.40). The mean hospice-level score for achieving these visits was 64.8% (median 70.2%; IQF 53.0%-80.9%). The Pearson’s correlation coefficients between hospice-level rates of visits at the end of life and the caregiver percentages for “definitely” recommending the hospice was 0.2418 and for rating the hospice a 9 or 10 on a 10-scale was 0.2587. These findings demonstrate significant variability across hospice providers and signal a positive correlation with caregivers’ quality ratings. Future work is needed to monitor the provision of these visits.

